# Effect of Weekly Antibiotic Round on Antibiotic Use in the Neonatal Intensive Care Unit as Antibiotic Stewardship Strategy

**DOI:** 10.3389/fped.2020.604244

**Published:** 2020-12-15

**Authors:** Bo Wang, Geng Li, Fei Jin, Jingwen Weng, Yaguang Peng, Shixiao Dong, Jingyuan Liu, Jie Luo, Hailan Wu, Yanhua Shen, Yao Meng, Xiaoling Wang, Mingyan Hei

**Affiliations:** ^1^Neonatal Center, Beijing Children's Hospital, Capital Medical University, Beijing, China; ^2^Neonatal Center, National Center for Child Health, Beijing, China; ^3^Center for Clinical Epidemiology and Evidence-Based Medicine, Beijing Children's Hospital, Capital Medical University, Beijing, China; ^4^Department of Clinical Pharmacy, Beijing Children's Hospital, Capital Medical University, Beijing, China

**Keywords:** antibiotic stewardship, antibiotic use, neonatal intensive care unit, hospitalization, children's hospital

## Abstract

**Background:** Antibiotics are commonly used in the neonatal intensive care unit (NICU). The objective was to observe the effect of weekly antibiotic round in NICU (WARN) to the antibiotic use in NICU.

**Methods:** A retrospective observational study was performed. Departmental-level diagnosis categories and the parameters of antibiotic usage in NICU for the period of 2016-2017 (Phase 1) and 2018-2019 (Phase 2) were collected. WARN in NICU was started since January 2018. A time series forecasting was used to predict the quarterly antibiotic use in Phase 2, based on data from Phase 1. The actual antibiotic use of each quarter in Phase 2 was compared with the predicted values.

**Results:** Totally 9297 neonates were included (4743 in Phase 1, 4488 in Phase 2). The composition of the disease spectrum between Phase 1 and Phase 2 was not different (*P* > 0.05). In Phase 1 and Phase 2, the overall antibiotic rate was 94.4 and 74.2%, the average accumulative defined daily dose per month was 199.00 ± 55.77 and 66.80 ± 45.64, the median antibiotic use density per month was 10.31 (9.00-13.27) and 2.48 (1.92-4.66), the median accumulative defined daily dose per case per month was 0.10 (0.09-0.13) and 0.03 (0.02-0.47), the number of patients who received any kind of antibiotic per 1000 hospital days per month was 103.45 (99.30-107.48) and 78.66 (74.62-82.77), rate of culture investigation before antibiotics was 64 to 92%, respectively, and all were better than the predicted values (*P* < 0.01).

**Conclusion:** The implementation of periodical antibiotic rounds was effective in reducing the antibiotics use in the NICU.

## Introduction

Empiric antibiotic therapy is common in neonatal intensive care units (NICUs) while neonatologists await the culture results, because the clinical signs of sepsis are subtle in neonates (1) and there is a lack of consensus regarding antibiotic usage (2). It has been found that despite negative blood cultures at 48 h, 24% of asymptomatic neonates born to chorioamniotitis mothers still received antibiotics for more than 48 h (3). Furthermore, there has been a 40-fold variation in antibiotic use in NICUs (2). Antibiotic stewardship is challenging in NICUs. Strategies and tools have been developed to improve appropriate antibiotic prescription, such as the electronic health record system for preventing inadvertent prolonged antibiotic duration (4), and the implementation of an automatic 48-h antibiotic stop order in the NICU (5). Patient-driven individualized antibiotic therapy in the NICU is pivotal, which largely depends on physicians' bedside close observation and therapeutic determination. The objective of this study was to develop a stewardship strategy for antibiotics by implementing a weekly antibiotic round in the NICU (WARN), and to observe the effect of this strategy on the appropriate use of antibiotics in the NICU.

## Materials and Methods

### Settings

The study was performed at Beijing Children's Hospital of Capital Medial University, the National Medical Center of Child Health, China. There were 60 beds in the level III NICU staffed by 20 neonatologists and 60 registered NICU nurses. All patients were out-born. The NICU recommended for all mothers to give maternal breast milk to their hospitalized infants, and no donor milk was used.

Daily bedside rounds were conducted by the attending neonatologists. A NICU medication handbook that was developed by the neonatologists and pharmacist based on textbooks and the current consensus was used as the working brochure for all physicians, residences, fellows, and pharmacists.

### Study Design and Implementation of WARN

This was a single-center retrospective observational study, randomization was not performed. The study period was from January 1st, 2016 to December 31, 2019 (January 2016 to December 2017 was considered to be Phase 1, and January 2018 to December 2019 was considered to be Phase 2). There was no exclusion criteria and all neonates admitted in the NICU were included. The study was not blinded and was approved by the Institutional Review Board of the hospital. Individual patient information was de-identified, and there was no need to obtain written consent from the parents.

The WARN was started in the first week of January 2018, in a weekly period. In detail, the antibiotic round was performed in each Monday afternoon for 1~1.5 h, the departmental physician-in-chief led the round, a fixed group of six attending staff neonatologists and one pharmacist attended the round each time, as well. All patients who were on antibiotics, including anti-bacterial agents and anti-fungal agents, were reviewed individually and patients who were not on antibiotics were not reviewed in the antibiotics round. For each antibiotic round, a senior attending physician was assigned to collect and report the patient's name, bed number, gestational age, age at admission (d), main diagnosis, criteria for using antibiotics, kinds of antibiotics, and plan of antibiotic course. He/she also reported the total antibiotic usage of the whole NICU as the ratio of patients who were on antibiotics, ratio of patients who were on a single kind of antibiotics, ratio of patients who were on two, three, or above kinds of antibiotics, the percentage of patients who had no antibiotics in the first 48 h after admission, the spectrum of antibiotics used, and the names of the top three antibiotics used. A summary note of the antibiotic round was also recorded by this senior attending doctor. A discussion was raised by the leading physician-in-chief for reasons of antibiotics use, kinds and compositions of antibiotics, plans for the antibiotic courses, and special issues related to the use of antibiotics for each patient. The final decision regarding the use of antibiotics for an individual patient was made on the basis of group discussion.

A research assistant was assigned to collect the following data at the departmental level from the electronic history of in-hospitalization system in a monthly period: total number of patients admitted and discharged, total patients days, length of stay in hospital, main diagnosis, antibiotic use rate (AUR), antibiotic use density (AUD), number of patients who received any kinds of antibiotics per 1,000 hospital days, accumulative daily defined dose (DDD) per case, pediatric conserve antibiotics (pCAs), number of hospital bed turnover and rate of culture investigation before antibiotics. The definitions of these metrics were listed in Table 1 (6–13). The microbiological investigations included blood culture, urine culture and cerebrospinal fluid culture (The cerebrospinal fluid culture were completed if the patient needed a full septic workup).

**Table 1:** Definitions of metrics.

**Metrics**	**Definition**
Total patient days	The number of patients who were treated the same period × Average days in hospital (6 ).
Length of stay in hospital (LOS, d)	The number of days between the admission date to hospital and the date of discharge from (7).
Antibiotic use rate	The proportion of patients received at least one kind antibiotic therapy to the total number of patients discharged (8).
Daily defined dose (DDD)	The international standard unit of measurement and is a measure of the average maintenance dose per day for a drug (9).
The accumulative DDD (DDDs)	The sum of DDD of all drugs used. The formula for calculating DDDs is: DDDs = ∑(total consumption of a specific drug (g) / DDD of the specific drug) (6).
Antibiotic use density	DDDs *100/ (The number of patients who were treated the same period*Average days in hospital) (6).
Rate of culture investigation before antibiotics	The number of culture (blood culture, CSF or urine culture if clinically necessary) completed before any antibiotics/Total number of patients who received antibiotic therapy*100% (10).
Hospital bed turnover	The total number of patients discharged divided by the average number of hospital bed provided, which is associated with the spectrum and severity of diseases (11).
Pediatric conserveantibiotics	Carbapenems, Glycopeptides and Linezolids were considered to be the watch and reserved group of antibiotics (12, 13).

### Statistical Analysis

This was not a randomized study, and the sample size was not estimated. The intervention was WARN, and the Phase 1 and Phase 2 cohorts were selected based on the intervention start time. To compare baseline data and antibiotic consumption between the Phase 1 and Phase 2 cohorts, the χ*2* test was used to compare differences in categorical variables, the two-sample *t*-test was used to compare parametric continuous variables, and the Mann–Whitney *U* test was used to compare non-parametric continuous variables. A *P* value < 0.05 was considered to be statistically significant, odds ratio (OR) and 95% confidence interval (95% CI) for categorical variables, mean or median difference and 95%CI for parametric or non-parametric continuous variables were also shown. Then, the metrics used to assess the antibiotic consumption running chart demarcated by season (3 month/each season) were generated. The Time Series Forecasting method was used to predict the antibiotic consumption of each quarter in Phase 2, based on the data from Phase 1. The comparison of the predicted and observed values was based on the results of the analysis. Exponential smoothing was used in the stationary sequence, and the autoregressive integrated moving average model (ARIMA) was used in the non-stationary sequence. All statistical analyses were conducted using SPSS version 23.0 (IBM, USA) and SAS version 9.4 (SAS Institute Inc., Cary, NC).

## Results

### Patient Categories in Phase 1 and Phase 2

Totally 9297 neonates were included (4743 in Phase 1, 4488 in Phase 2). There was no statistically significant difference in the composition of the disease spectrums between the two Phases (*P* > 0.05, Table 2). There was also no statistically significant differences in the ratio of the number of cases with infectious disease to the total number of admission (OR = 1, 95%CI 0.92 to 1.11, *P* > 0.05), total number of discharge (mean difference 10.63, 95%CI−0.18 to 21.43, *P* > 0.05), total patient days (mean difference 56.83, 95% CI−11.02 to 124.69, *P* > 0.05), the median length of stay in hospital per patient (median difference−0.32, 95%CI−0.77 to 0.13, *P* > 0.05), or the number of hospital bed turnover (mean difference 1.69, 95%CI−2.22 to 5.61, *P* > 0.05) between Phase 1 and Phase 2 (Table 2).

**Table 2:** Baseline demographic and clinical characteristics between Phase 1 and Phase 2 cohorts.

**Characteristics**	Phase 1^*^	Phase 2^*^	***P* value**
First diagnosis of discharge, *n* (%)			
Sepsis/Suspectedsepsis	417 (8.79%)	356 (7.93%)	
Neonatal meningitis	131 (2.76%)	125 (2.79%)	
Neonatal pneumonia	535 (11.28%)	536 (11.94%)	
Hyperbilirubinemia	1006 (21.21%)	896 (19.96%)	
Surgical intervention	64 (1.35%)	76 (1.69%)	0.113^#^
IVH	231 (4.87%)	187 (4.17%)	
HIE or Convulsions	866 (18.26%)	885 (19.72%)	
Others	1439 (30.34%)	1427 (31.80%)	
Infectious diseases at discharge, *n* (%)	1083 (22.8%)	1017 (22.7%)	0.843
Total number of admission per month, n^**^	197.63 ± 20.68	187 ± 16.23	0.054
Total patient days per month, d^**^	1820.96 ± 125.31	1764.13 ± 107.58	0.099
Median length of stay in hospital, d^&^	9.13 (8.73-9.43)	9.53 (8.81-10.00)	0.132
Hospital bed turnover per month, times^**^	97.97 ± 8.26	96.28 ± 4.75	0.389

*HIE, hypoxia ischemia encephalopathy; IVH, Intracranial ventricular hemorrhage*.

* *Phase 1 was from January, 2016 to December, 2017, and Phase 2 was from January, 2018 to December, 2019*.

# *The P value for the difference of diagnosis composition in each Phase*.

** *Data presented as mean ± SD*.

& *Data presented as median (Inter Quartile Range, IQR, Q25 – Q75)*.

### Comparison of Antibiotic Consumption Between Phase 1 and Phase 2

The antibiotic consumption parameters are listed in Table 3. In the Phase 1, there were 4478 of 4743 (94.4%) neonates who received antibiotics. In Phase 2, there were 3338 of 4488 (74.2%) neonates who received antibiotics (OR = 5.89, 95%CI 5.12 to 6.78, *P* < 0.01). Both the antibiotics use rate (Figure 1A) and the number of patients received any kinds of antibiotics per 1000 hospital days (Figure 1B) were significantly reduced in Phase 2 (*P* < 0.05). After WARN in Phase 2, the mean DDDs per month significantly reduced by 66% (mean difference 132.16, 95%CI 102.55 to 161.77, *P* < 0.01), the median AUD per month significantly decreased by 76% (median difference 7.13, 95%CI 6.11 to 8.44, *P* < 0.01), and the median DDDs per case and number of patients who received any kind of antibiotic per 1,000 hospital days every month reduced by 70% (median difference 0.07, 95%CI 0.06 to 0.08, *P* < 0.01) and 24% (median difference 25.05, 95%CI 21.32 to 28.75, *P* < 0.01,), respectively. Rate of culture investigation before antibiotics was increased from 62% in Phase 1 to 92% in Phase 2 (OR = 0.148, 95%CI 0.13 to 0.17, *P* < 0.01). In Phase 1 and Phase 2, the pCA use rates were 4.2 and 4.9%, respectively (OR = 0.85, 95%CI 0.69 to 1.03, *P* > 0.05). There was no statistically significant difference in the number of patients who received pCAs therapy per 1,000 hospital days between Phase 2 and Phase 1 (mean difference−1.01, 95%CI−2.09 to 0.07, *P* > 0.05).

**Table 3:** Antibiotic consumption between Phase 1 and Phase 2 cohort.

**Characteristics**	Phase 1^*^	Phase 2^*^	***P* value**
Antibiotics use rate, % (*n*)	94.4% (4478)	74.2% (3328)	<0.01
Pediatric conserve antibiotics use rate, % (*n*)	4.2% (197)	4.9% (219)	0.093
Rate of culture investigation before antibiotics, % (*n*)	64% (2854)	92% (3069)	<0.01
Rate of culture investigation before conserve antibiotics, % (*n*)	99% (195)	98.6% (216)	0.74
AUD	10.31 (9.00-13.27)	2.48(1.92-4.66)	<0.01
DDDs^**^	199.00 ± 55.77	66.80 ± 45.64	<0.01
DDDs per case	0.10 (0.09-0.13)	0.03 (0.02-0.47)	<0.01
Number of patients who received any kinds of antibiotics per 1000 hospital days, *n*^#^	103.45 (99.30-107.48)	78.66 (74.62-82.77)	<0.01
Number of patients who received pediatric conserve antibiotics per 1000 hospital days, n^**^	4.56 ± 2.04	5.57 ± 1.65	0.066

*AUD, Antibiotic use density; DDD, Defined daily dose*.

* * Phase 1 was from January, 2016 to December, 2017, and Phase 2 was from January, 2018 to December, 2019*.

** *Data presented as mean ± SD*.

# *Data presented as median (Inter Quartile Range, IQR, Q25—Q75)*.

**Figure 1:**
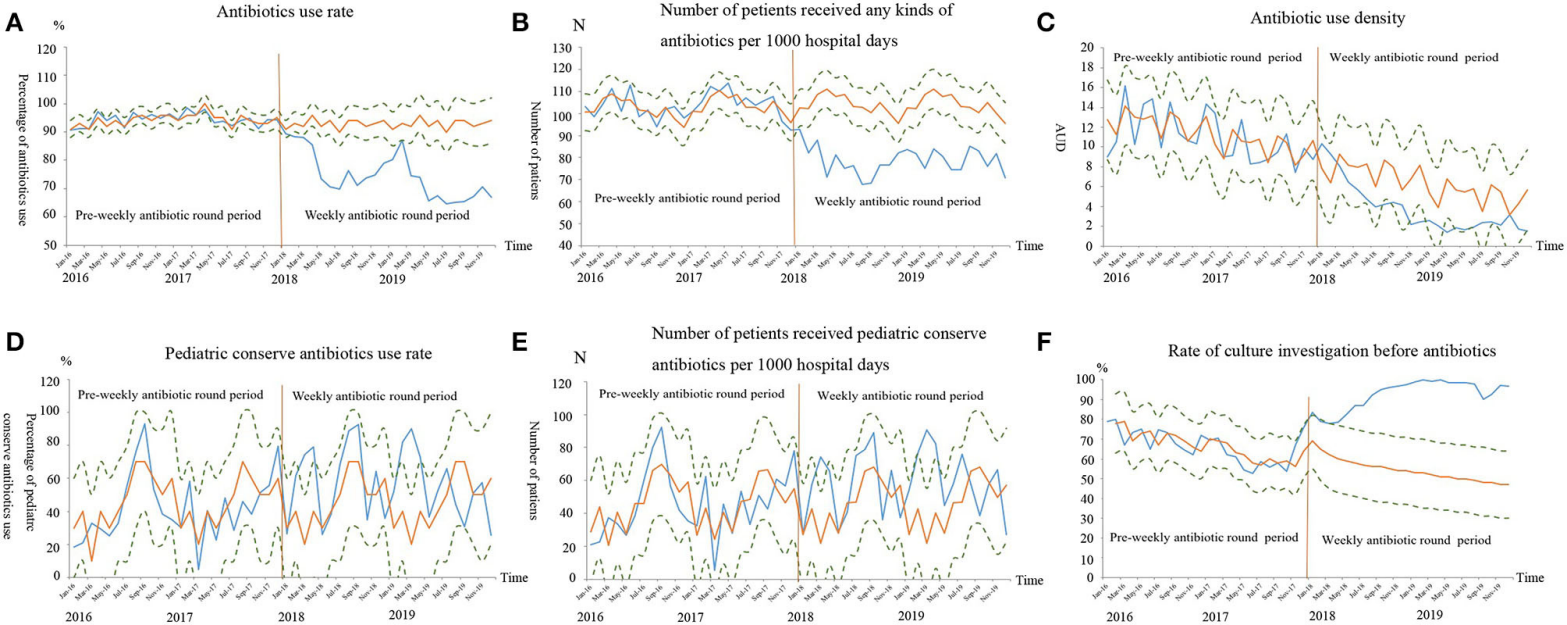
The antibiotics consumptions after time series forecasting. The blue curve was the observed values curve from January 2016 to December 2019, the yellow curve was the predicted time-series values curve based on the data from January 2016 to December 2017, and the dots curves were the upper/lower level of 95% confidence interval of the predicted curve. The orange vertical line indicated the starting time of the weekly antibiotic round. The observed values curve for antibiotics use rates **(A)**, number of patients who received any kind of antibiotic per 1,000 hospital days **(B)**, antibiotic use density **(C)**, had an overall decreasing trend from January 2016 to December 2019, but were progressively lower than the predicted values curve from January 2018 to December 2019 (*P* < 0.01). The pediatric conserve antibiotics rates **(D)** and number of patients who received pediatric conserve antibiotics per 1000 hospital days **(E)** were not significantly different between the observed values curve and predicted time-series values curve (*P* > 0.05). The observed values curve for rate of culture investigation before antibiotics **(F)** had an overall increasing trend from January 2016 to December 2019, but were progressively higher than the predicted values curve from January 2018 to December 2019 (*P* < 0.01).

### Comparison of Observed and Predicted Antibiotic Consumption in Phase 2

The auto-regressive test indicated that rate of culture investigation before antibiotics was a non-stationary sequence, while the others were stationary sequences. In Phase 2, the observed overall AUR and the number of patients who received any kinds of antibiotics per 1,000 hospitals day values were all lower than their predicted values and their observed value curves were all outside the lower limit of the 95% CI of the predicted curves. In Phase 2, although decreased trend was observed in AUD, its observed value curve was within the 95% CI of the predicted value curve (Figure 1C). For the pCAs AUR and number of patients who received pCAs therapy per 1,000 hospital days in each quarter, the observed curves were basically consistent with the predicted values and were located within the 95% CI of the predicted value curves (Figures 1D,E).

The ARIMA model (1, 1, 1) was used to fit rate of culture investigation before antibiotics per quarter in Phase 2. The observed values were all higher than the predicted values and the observed value curve was beyond the upper limit of the 95% CI of the predicted value curve (Figure 1F).

## Discussions

To the best of our knowledge, this is the first report on the effect of antibiotic round on antibiotic use in the NICU as an antibiotic stewardship strategy. This study found that the WARN could reduce the antibiotic use rate and antibiotic consumption, and increase rate of culture investigation before antibiotics. Furthermore, the time series forecasting showed that the actual antibiotic consumption values were better than the predicted values based on the data before WARN.

Worldwide, a considerable proportion of antibiotics are used in non-infection cases in NICUs. A survey in the United States reported that about 50% of NICUs with zero rates of culture-proven infection had the highest antibiotic use quartile, in 127 NICUs during 2013 (2). Fjalstad et al. found that almost 26% of admitted neonates in NICUs received antibiotics without being diagnosed with infection from 2009 to 2011 in Norway (14). A study in India reported that the percentage of neonates who received antibiotics was up to 89%, including patients with no infection or unclear infection (15). Our own data from to 2016–2017 revealed a similar possible over-use of antibiotics when the antibiotic use rate was 94.4%, but the percentage of patients who were diagnosed with infection was far less. The major reasons for administering antibiotics to noninfectious neonates were as follows: (1) The clinical pictures of infectious and non-infectious neonates often overlapped (e.g., respiratory distress within 24 h of birth, transient tachypnea, feeding difficulties, etc.) (16), which cause neonatologists to be unable to fully differentiate non-septic neonates from septic neonates. It was almost a clinical routine in NIUCs to administer empiric antibiotics for 48 h while awaiting culture results. (2) It was quite often that some neonatologists would continue to use antibiotics longer than 48 h, even though the cultures were negative with respect to culture-negative sepsis (17). (3) There was a lack of fixed NICU stewardship policies, or a fixed team of senior attending neonatologists who were focusing on antibiotic use. The positive results of the WARN study indicated that fixing a team of senior neonatologists and pharmacists to routinely perform weekly antibiotic surveillance is an easy and practical way for NICU to effectively decrease antibiotic use.

The time series forecasting results showed that the actual antibiotic use after the implementation of WARN was consistently lower than the predicted values based on the data before WARN. This indicated a positive effect of WARN to help further decrease antibiotic use, in addition to other quality improvement strategies for antibiotic management. Other strategies for antibiotic stewardship have also been reported previously. For example, a neonatal sepsis calculator in an Australian perinatal referral center has been developed to reduce the number of neonates ≥35 weeks who require empirical antibiotics for suspected EOS and did not result in the late presentation of EOS or treatment delay (18), and it was estimated that antibiotic use could be reduced in a large number of neonates in the USA by applying the calculator (19). Lamba et al. showed that the antibiotic level for late onset sepsis was appropriately de-escalated by implementing the evaluation of a multidisciplinary antimicrobial stewardship team (20). Cantey et al. showed that the number of days of antibiotic therapy per 1,000 patient-days was reduced by limiting the duration of pneumonia and culture-negative sepsis to five days and setting discontinuing empirical therapy after 48 h in electronic medical records, which did not cause adverse outcomes (21). Tolia VN reported that setting an automatic stop order at 48 h for antibiotics could reduce the percentage of infants who receive antibiotics > 48 h and antibiotic days of therapy during very low birth weight infants first seven days of life (22). Thampi N et al. reported the approach of daily prospective audit and feedback as antibiotic stewardship decreased antibiotic use days of therapy per 1,000 patient-days (23). Although different forms of antibiotic stewardship have been developed in NICUs, each strategy had its limitation; thus, a better antibiotic stewardship strategy is still needed. The WARN in our study was implemented by a fixed group of senior neonatologists, including an NICU pharmacologist, and was led by the departmental head. This stewardship method is probably a better way to ensure more appropriate antibiotic usage. The final decision to use antibiotics for each patient was made on the basis of a group discussion, which could avoid the risk of inadvertent discontinuation of necessary antibiotic therapy. The same as the approach of prospective audit and feedback, antibiotic stewardship includes a fixed group of attending staff, however, the period of WARN was a week, which provided enough time for attending physician to collect the data, and we documented the content of advices, which could help review the change of prescribing practices. But the difference was mainly the time interval that Thampi et al. had the audit and feedback daily and WARN was held once per week.

The mean DDDs per month in our study were significantly reduced by 66%, and the median DDDs per case and the AUD in the previous month also significantly decreased after WARN, by 70 and 76%, respectively. Similar to our results, Astorga et al. reported that the total antibiotic doses given per patient and doses per patient-day were reduced by an automatic 48-h antibiotic stop order in electric health records (5). A study in a rural hospital NICU in India showed that DDD per 100 patient-days of third-generation cephalosporin was reduced by an antibiotic policy on sepsis (24). Although the result of the time series forecasting showed that the observed value curve of AUD after WARN was all within the 95% confidence interval of its predicted value curve, based on data before WARN, a decreasing trend was apparent in the observed value curve. The reason for the negative result in the time series forecasting may be that the result was only fit to a model based on data before WARN, which may not accurately predict all situations. Moreover, there was a large difference between the lower and upper 95% confidence intervals of the predicted value curve. The lower limit of the 95% confidence interval of the predicted value curve gradually approached zero since January, 2019, and the actual result could not exceed the predicted interval.

A study from an NICU in Poland revealed that the antibiotic consumption of bloodstream infection confirmed by microbiological test was less than that of non-confirmed bloodstream infections in very low birth weight neonates (25). Thus, timely identification of the source of infection by microbiological testing can reduce antibiotic consumption. Rate of culture investigation before conserve antibiotics in our study did not change significantly in both Phase 1 and Phase 2, while the total rate of culture investigation before antibiotics was higher in Phase 2, and the observed value curve was beyond the upper limit of the 95% confidence interval of the predicted value curve. This indicated that the positive effect of WARN enhances the concept of culture-before-using-antibiotics in NICU infants. The composition of disease spectrum and proportion of infectious diseases between Phase 1 and Phase 2 did not significantly differ. According to previous studies on antibiotic stewardship that studied the judicious use of meropenem and vancomycin (26, 27), the use of pCAs (carbapenem, linezolid, and glycopeptide) were used to evaluate the proportion of patients with serious infections. There was no significant difference in the rate of pCA use and the number of patients who received pCAs therapy per 1,000 hospital days between the two Phases.

The limitations of this study were as follows: (1) It was a retrospective single-center study. (2) The study period only included the two years before the implementation of WARN and the 2 years of WARN implementation. (3) The effect of WARN was not investigated in any specific antibiotics subgroup (such as the third-generation cephalosporin) or any specific infectious disease subgroup. (4) The days of therapy (DOT) or length of therapy (LOT) per agent were not used in this study, both of which are commonly used metrics within the pediatric population. In children, antibiotic dosing is based on body weight. As such, in order to calculate a pediatric DDD, an average body weight for the pediatric population would need to be assumed. Given there is also a large variation in body weight within this population, the question remains whether DDD is the most adequate metric to quantify antibiotic use.

In summary, the implementation of periodical antibiotic rounds provide an effective strategy for reducing overall antibiotic use in NICU neonates. WARN effectively reduced the overall antibiotic use in the NICU and provides a practical way to achieve more appropriate antibiotic use in the NICU.

## Data Availability Statement

The original contributions presented in the study are included in the article/supplementary materials, further inquiries can be directed to the corresponding author/s.

## Ethics Statement

The study was approved by the Ethics Committee of Beijing Children's Hospital, Capital Medical University. Written informed consent from the participants' legal guardian/next of kin was not required to participate in this study in accordance with the national legislation and the institutional requirements.

## Author Contributions

BW and MH conceptualized and designed the study, drafted the initial manuscript, and reviewed and revised the manuscript. GL, FJ, JW, SD, JLi, JLu, HW, YS, YM, and XW designed the data collection instruments, collected data, and reviewed and revised the manuscript. YP carried out the initial analyses. MH contributed to the conception and design of the work, and revised it critically for important intellectual content. All authors approved the final manuscript as submitted and agree to be accountable for all aspects of the work in ensuring that questions related to its accuracy or integrity are appropriately investigated and resolved.

## Conflict of Interest

The authors declare that the research was conducted in the absence of any commercial or financial relationships that could be construed as a potential conflict of interest.

## Abbreviations

ARIMA: autoregressive integrated moving average model
AUD: antibiotic use density
AUR: antibiotic use rate
95% CI: 95% confidence interval
DDD: defined daily dose
DDDs: accumulative DDD
EOS: early-onset sepsis
LOS: late-onset sepsis
NICU: neonatal intensive care unit
pCAs: pediatric conserve antibiotics
WARN: weekly antibiotic round in NICU.
